# Association between breastfeeding and osteoporotic hip fracture in women: a dose-response meta-analysis

**DOI:** 10.1186/s13018-019-1541-y

**Published:** 2020-01-16

**Authors:** Haixiang Xiao, Quan Zhou, Gouqi Niu, Guansheng Han, Zhongchuan Zhang, Qingbo Zhang, Jianzhong Bai, Xunbing Zhu

**Affiliations:** 1 Department of Orthopedics, Jingjiang People’s Hospital, Jingjiang, 214500 China; 2 Department of Orthopedics, The Second Affiliated Hospital of Bengbu Medical College, Bengbu, 233000 China; 3 Department of Science and Education, First People’s Hospital of Changde City, Changde, 415003 China

**Keywords:** Hip fractures, Breast feeding, Meta-analysis

## Abstract

**Objective:**

Approximately 300 mg of calcium a day is provided into infants to maintain the physical development of infants, and 5 to 10% bone loss occurs in women during breastfeeding. Hip fractures are considered the most serious type of osteoporotic fracture. We performed this meta-analysis to investigate the association between breastfeeding and osteoporotic hip fractures.

**Material and methods:**

PubMed and Embase were searched until May 1, 2019, for studies evaluating the relationship between breastfeeding and osteoporotic hip fracture in women. The quality of the included studies was evaluated by the methodological index for non-randomized studies (MINORS). For the dose-response meta-analysis, we used the “generalized least squares for trend estimation” method proposed by Greenland and Longnecker to take into account the correlation with the log RR estimates across the duration of breastfeeding.

**Results:**

Seven studies were moderate or high quality, enrolling a total of 103,898 subjects. The pooled outcomes suggested that breastfeeding can decrease the incidence of osteoporotic hip fracture (RR = 0.64 (95% CI 0.43, 0.95), *P* = 0.027). Dose-response analysis demonstrated that the incidence of osteoporotic hip fracture decreased with the increase of breastfeeding time. The RR and 95% CI for 3 months, 6 months, 12 months, and 24 months were RR = 0.93, 95% CI 0.88, 0.98; RR = 0.87, 95% CI 0.79, 0.96; RR = 0.79, 95% CI 0.67, 0.92; and RR = 0.76, 95% CI 0.59, 0.98, respectively, whereas no significant relationship was found between them when the duration of breastfeeding time was more than 25 months.

**Conclusions:**

Our meta-analysis demonstrated that the incidence of osteoporotic hip fracture decreased with the extension of breastfeeding time. However, there is no significant relationship between them when the duration of breastfeeding time was more than 25 months.

## Introduction

Osteoporotic fracture has become a common health problem all over the world due to the aging of the population [1]. More than one third of 50-year-old women will suffer from serious osteoporotic fractures in their remaining lifetimes [2], and every 3 s one osteoporotic fracture occurs somewhere in the world. Hip fracture is also known as the last fracture in their remainder lifetimes and is considered the most serious type of osteoporotic fractures due to high morbidity and mortality. The previous study demonstrated that an approximated 20 to 40% of patients with hip fracture will suffer from death in 1 year, and only one in three of these patients can recover their previous functional status [3].

Breastfeeding is an essential reproductive function among females, provides approximately 300 mg of calcium a day into infants to maintain the physical development of infants [4], and results in 5 to 10% bone loss in women during breastfeeding [5]. Besides, they need to provide large amounts of calcium for mineralization of the fetal and neonatal skeleton [6]. Therefore, bone loss in women starts earlier than in men [7], and this may be one reason why osteoporosis occurs in older women far more than in men. Women with severe osteoporosis may suffer from a fragility fracture [8]. However, it is controversial whether breastfeeding can increase the risk of osteoporotic hip fractures, and the possible mechanism is considerably complicated. Some studies demonstrated breastfeeding may contribute to protection against osteoporotic hip fractures [9–12], and the risk will decrease with the extent of breastfeeding [9, 10, 12]. However, some studies indicated that prolonged breastfeeding time can decrease bone mineral density [13, 14]. Whereas others indicated that breastfeeding was unrelated to osteoporotic hip fracture or bone density in postmenopausal women [15, 16]. What is more, there is insufficient evidence in individual studies to show the relationship between them and express conflicting conclusions.

Therefore, we performed this dose-response meta-analysis to investigate the association between breastfeeding and osteoporotic hip fracture in women, hypothesizing that breastfeeding is associated with lower osteoporotic hip fracture, and to provide a better guiding strategy for clinicians.

## Materials and methods

We carried out this meta-analysis according to the Preferred Reporting Items for Systematic Reviews and Meta-Analysis (PRISMA) statement [17] and the Cochrane Collaboration guidelines strictly.

### Search strategy

PubMed and Embase were searched until May 1, 2019. Besides, we manually searched the reference lists of all identified relevant publications to find potential studies, without language restrictions. Search terms included the keywords related to osteoporosis, hip, fracture, breastfeeding, and their variants. The Boolean operators were used to combine them.

### Study selection and eligibility criteria

Inclusion criteria: (1) the exposure was breastfeeding, (2) the outcome was osteoporotic hip fracture, (3) sample size was more than 100, and (4) the included article provides sufficient data. Exclusion criteria: (1) other types of fractures; (2) hip fracture due to severe trauma; (3) case report, review, commentary, and study just included an abstract; and (4) there was duplicate publication

### Data extraction

Data extraction was performed by two reviewers. General characteristics of the patient were extracted from included studies: author, year of publication, study designs, sample size, age, country, diagnostic methods, and adjustment for covariance. Any disagreements were resolved by discussion to reach a consensus. All extracted data were entered into a predefined standardized Excel (Microsoft Corporation, USA) file carefully.

### Quality assessment

Two reviewers evaluated the quality of studies by the methodological index for non-randomized studies (MINORS) respectively. The MINORS is a useful tool for assessing the quality of non-randomized studies [18]. This scale contains 12 items. Each index is 2 scores, the total score is 24, and higher scores indicate higher quality.

### Statistical analysis

Statistical analyses were performed using R software and STATA 12.0. The association between breastfeeding and osteoporotic hip fracture was expressed as RR with corresponding 95% CIs. *P* value less than 0.05 was regarded as statistically significant. Statistical heterogeneity was assessed by the *I*^2^ statistic; when *I*^2^ > 50% and *P* < 0.1, random effect model was used; if not, fixed effect model was used. Generic inverse variance was used in this meta-analysis. Dose-response analysis was performed using the method described by Greenland and Longnecker [19], which takes into account the correlation with the log RR estimates across the duration of breastfeeding. Only studies providing the cases and cohort size subjects and the RRs could be included in the analysis. The value assigned to each category of breastfeeding month was the median provided by the original study. For the studies not containing a median, we used the midpoint for closed category; when the highest category was open-ended, we assumed the midpoint of the category was set at 1.2 times the lower boundary [20]. We estimated the potential non-linear dose-response relationship in two stages: firstly, a restricted cubic spline model was estimated; secondly, the 2 regression coefficients and the variance matrix within each study were combined. *P* value for non-linearity was calculated by testing the null hypothesis that the coefficient of the second spline is equal to zero [21].

## Results

### Search result

**Fig. 1:**
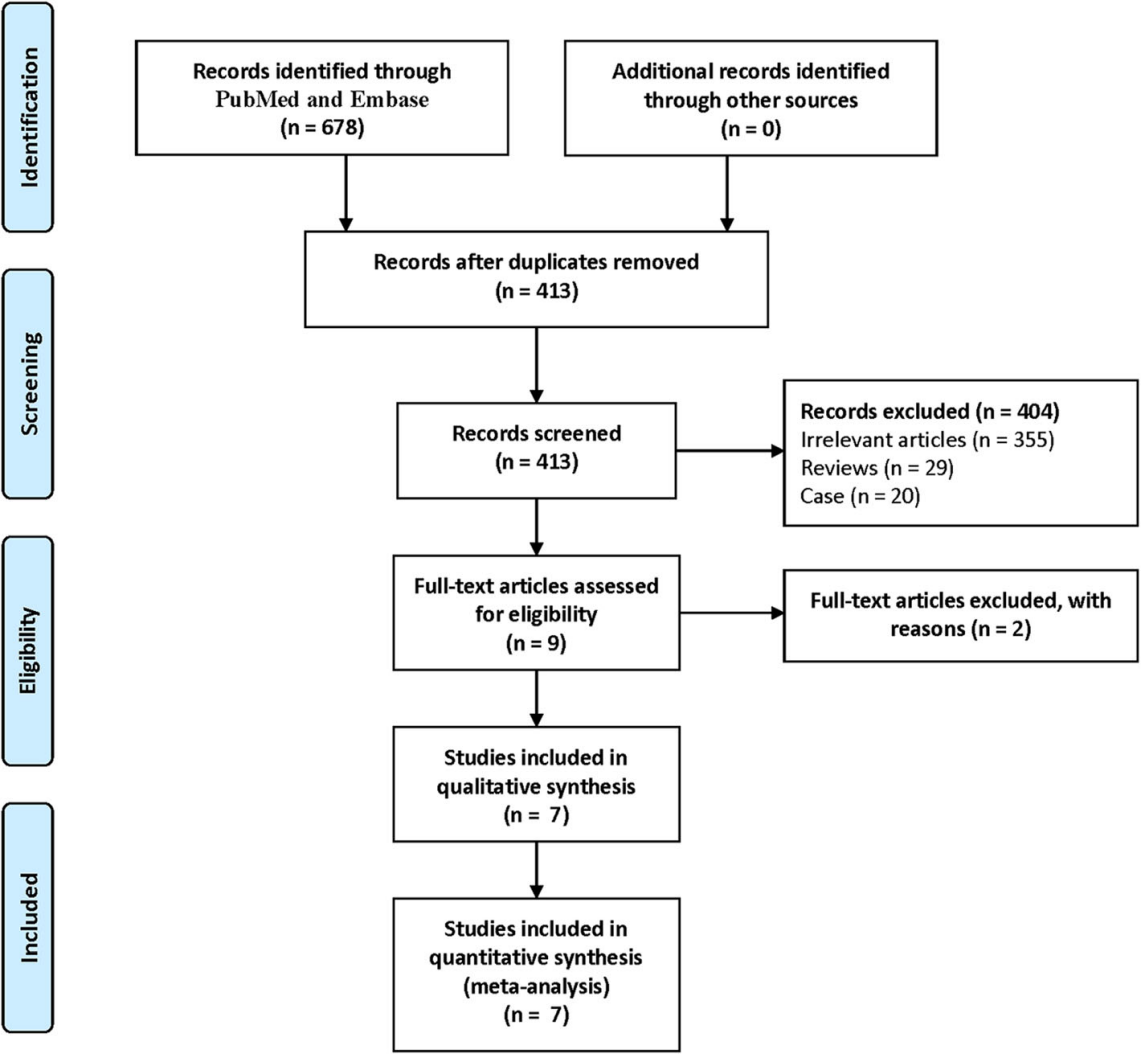
The flow chart of studies selection

A total of 678 potentially relevant references were founded. Endnote X8 (version 18.0.0.10063) was used to remove 265 duplicate studies. By scanning the titles and abstracts, 404 studies were excluded. After a scan of the full texts, 2 studies were excluded, which merely include one set of data for the follow-up period [22, 23]. Finally, 7 studies were included. Details of the study selection process were shown in Fig. 1.

### Study characteristics

Two of the seven were cohort studies [7, 15], and five were case-control studies [10–12, 16, 24]. These study sample sizes ranged from 308 to 92,980 subjects, with a total of 103,898 subjects. The main characteristics of the included trials are summarized in Table 1.

**Table 1:** Characteristics of included studies

Study (year)	Country	Age (year)	Sample size (test group)	Diagnostic method	Study design	Follow-up (year)	Adjustment for covariance
Alderman et al. [24] (1986)	USA	50–74	1520 (427)	No report	Case-control	1976–1980	Age, estrogen use, and relative weight
Hoffman et al. [16] (1993)	USA	≥ 45	348 (174)	Radiographs	Case-control	1987–1989	Age, hospital of recruitment, age group, and number of live births
Cumming et al. [12] (1993)	Australia	≥ 65	308 (174)	No report	Case-control	1990–1991	Age, body mass index, history of hormone therapy use, et..
Michaelsson et al. [11] (2001)	Sweden	50–81	4640 (1328)	No report	Case-control	1993–1995	Age, hormone therapy, parity, body mass index, etc.
Huo et al. [10] (2003)	China	≥ 50	354 (118)	Radiographs	Case-control	1994–1996	No report
Bjørnerem et al. [9] (2011)	Norway	50–94	3748 (335)	No report	Cohort	1974–2008	Age, height, body mass index, smoking, alcohol use, etc.
Crandall [15] (2017)	USA	50–79	92,980 (1185)	No report	Cohort	1993–2005	Age, race-ethnicity, smoking status, body mass index, etc.

### Quality assessment

The average score was 17.3 (range, 15–19), suggesting that all the studies were of moderate or high quality. Details of the quality assessment of included studies were shown in Table 2.

**Table 2:** The study designs and MINORS appraisal scores for included studies

Study	MINORs methodological criteria												Total
	1	2	3	4	5	6	7	8	9	10	11	12	
Alderman et al. [24]	2	2	0	2	0	2	2	0	2	2	1	2	17
Hoffman et al. [16]	2	2	0	2	0	2	2	0	2	2	1	2	17
Cumming et al. [12]	2	2	0	1	0	1	2	0	2	2	1	2	15
Michaelsson et al. [11]	2	2	0	2	0	2	2	0	2	2	1	2	17
Huo et al. [10]	2	2	0	2	0	2	2	0	2	2	1	2	17
Bjørnerem et al. [9]	2	2	2	2	0	2	2	0	2	2	1	2	19
Crandall et al. [15]	2	2	2	2	0	2	2	0	2	2	1	2	19

The MINORs criteria include the following items: (1) a clearly stated aim, (2) inclusion of consecutive patients, (3) prospective data collection, (4) endpoints appropriate to the aim of the study, (5) unbiased assessment of the study endpoint, (6) a follow-up period appropriate to the aims of the study, (7) less than 5% loss to follow-up, (8) prospective calculation of the sample size, (9) an adequate control group, (10) contemporary groups, (11) baseline equivalence of groups, and (12) adequate statistical analyses

The items are scored as follows: 0 (not reported), 1 (reported but inadequate), and 2 (reported and adequate)

### Clinical outcomes

**Fig. 2:**
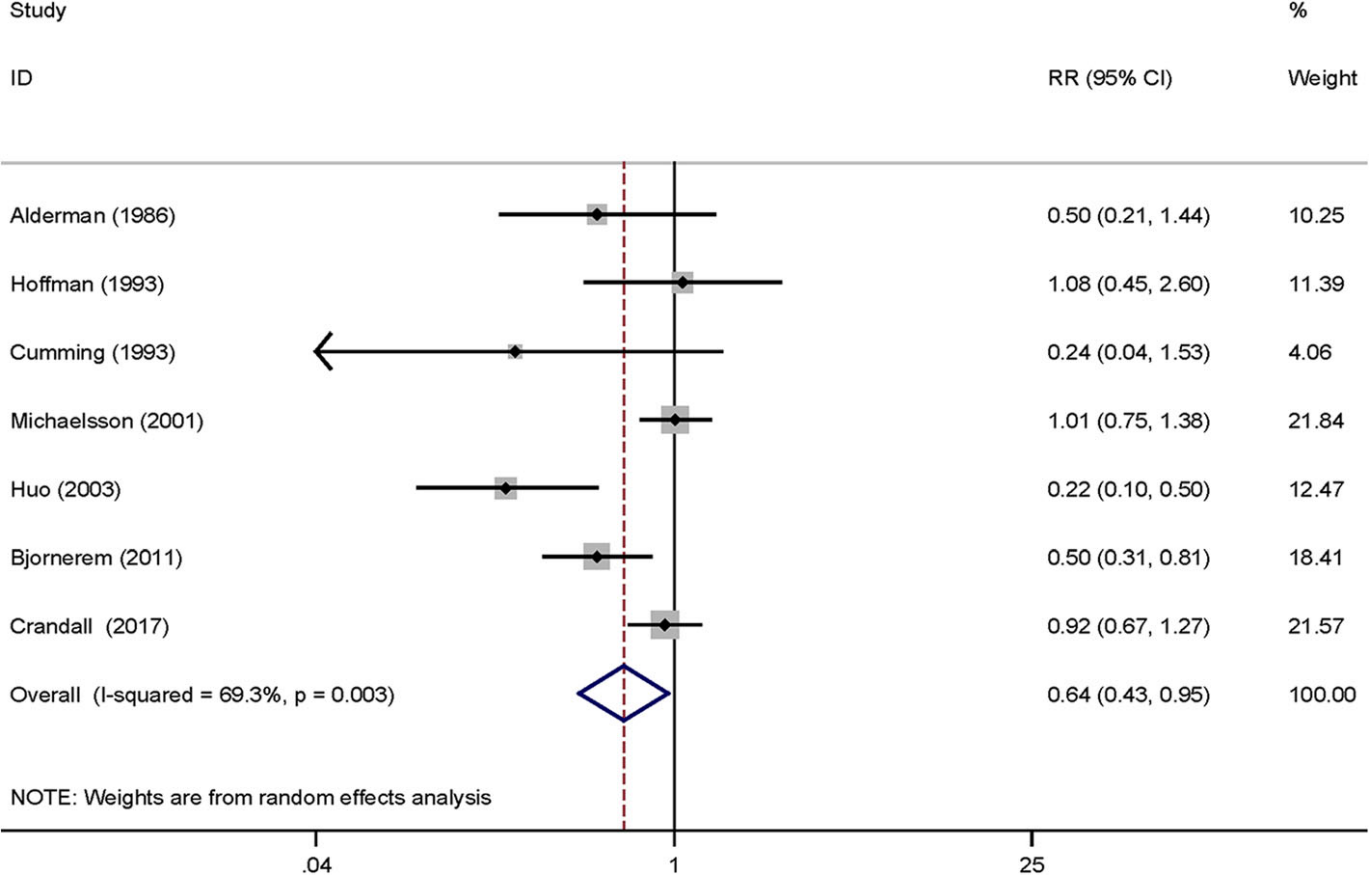
The forest plot for the effects of breastfeeding and non-breastfeeding groups on the incidence of osteoporotic hip fractures

Seven studies [7, 10–12, 15, 16, 24] provided available data and the pooled outcomes demonstrated that breastfeeding can decrease the incidence of osteoporotic hip fracture (RR = 0.64 (95% CI 0.43, 0.95), *P* = 0.027, *I*^2^ = 69.3%; Fig. 2).

### Dose-response analysis

**Fig. 3:**
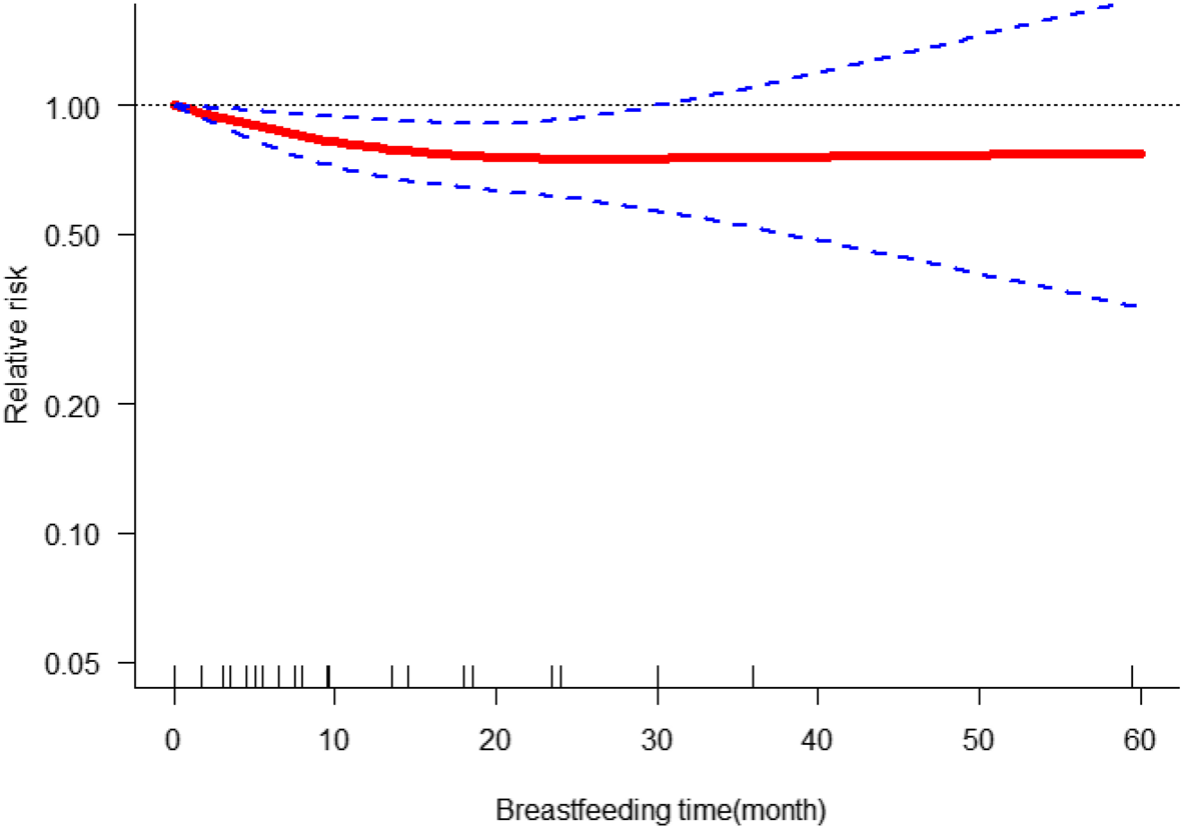
The dose-response analysis for the effects of breastfeeding on the incidence of osteoporotic hip fractures

Seven studies [7, 10–12, 15, 16, 24] provided continuous data on dose-response meta-analysis. Using a restricted cubic spline model, we observed a non-linear dose-response relationship between the duration of breastfeeding and risk of osteoporotic hip fracture (*χ*^2^ test for non-linearity = 9.7 (df = 2), *P* = 0.0079) and the heterogeneity among studies was not significant (multivariate Cochran *Q* test for heterogeneity = 19.9 (df = 12), *P* = 0.069, *I*^2^ = 39.6%); the dose-response curve (shown in Fig. 3) indicates that with the increase of breastfeeding time, the risk of osteoporotic hip fracture decreased gradually. With the females without breastfeeding as a reference group, the RR and 95% CI for 3 months, 6 months, 12 months, and 24 months were RR = 0.93, 95% CI 0.88, 0.98; RR = 0.87, 95% CI 0.79, 0.96; RR = 0.79, 95% CI 0.67, 0.92; and RR = 0.76, 95% CI 0.59, 0.98, respectively. When the duration of breastfeeding time was more than 25 months, there was no significant relationship between them.

## Discussion

### Main findings

This study demonstrated that there was a non-linear relationship between breastfeeding and osteoporotic hip fracture. The risk of osteoporotic hip fracture decreased with the extent of breastfeeding. However, there is no significant relationship between them when the duration of breastfeeding time was more than 25 months.

The association between breastfeeding and bone mineral density (BMD) in older women is complicated; studies in different regions have yielded different conclusions. In a nationwide survey in Korea by Hwang et al., the outcomes indicated that breastfeeding for more than 37 months can decrease the BMD in postmenopausal women [14]. Fox et al. pointed out there was no significant difference in the radius BMD between the women who was breastfeeding with those who were not [25]. Murphy et al. reported that there was no association between BMD and breastfeeding in the hip and spine [26], which is similar to the outcomes of Crandall et al. [15]. The study of Bjørnerem et al. demonstrated that the level of BMD of female breastfeeding for about 20 months or more were similar to those who did not breastfeed at the distal forearm and hip [9]. Besides, both Zhang et al. and Lenora et al. reported that breastfeeding does not significantly reduce BMD [27, 28]. What is more, the study of Melton et al. demonstrated that breastfeeding for more than 8 months was associated with higher BMD at the femur and spine [29]. Chantry et al. point out that compared to women who are not breastfeeding, lactation among adolescent mothers had a higher hip BMD at 20 to 25 years old [30].

It is controversial whether breastfeeding impacts on fractures due to inconsistent breastfeeding time. Bolzetta et al. found that breastfeeding more than 18 months significantly increases the risk of spinal fractures in menopausal women [31], similar to the study of Dursun et al. [32]. But Chan et al. considered that breastfeeding for 24 months or more was protective against vertebral fracture [33]. Bjørnerem et al. point out that there was no significant difference in wrist fracture between the breastfeeding groups and the non-breastfeeding group [7]. Whereas the study of Mallmin et al. demonstrated that the ever breastfeeding group has a lower risk of factors than the never breastfeeding group at the distal forearm [34]. The study of Hwang et al. suggested that prolonged breastfeeding has no significant effect on the incidence of osteoporotic hip fractures [14]. However, Bjørnerem et al. indicated that breastfeeding has no long-term deleterious effect on bone fragility and fractures, even reduced risk for hip fracture in menopausal women [9]. The outcomes of our meta-analysis demonstrated that breastfeeding within 25 months contribute to a reduced risk for osteoporotic hip fractures, when breastfeeding is longer than 25 months, there is no significant relationship between them.

### Heterogeneity analysis

Most of the included studies had adjusted for potential confounders and this meta-analysis was restricted to the patients with osteoporotic hip fracture, which were higher homogeneously and selective. However, the pooled results in Fig. 2 had high heterogeneity, so we made further investigation to find the source of heterogeneity. Heterogeneity remains high after the exclusion of each study once a time. Different follow-up time and countries may be the significant source of heterogeneity.

### Possible mechanisms

The main factors regulating BMD are Parathyroid hormone-related protein (PTHrP), estrogen, and other markers for bone formation during lactation. The secretion of PTHrP counterbalances bone loss during breastfeeding; PTHrP can stimulate reabsorption of calcium from the maternal skeleton, renal tubular reabsorption of calcium, and indirect suppression of parathyroid hormone (PTH) [6]. Estrogen deficiency will increase skeletal resorption and increase the blood calcium concentration, which increases renal calcium losses [35]. However, the concentration of estrogen increases during breastfeeding, which promotes bone formation to a certain extent [36]. During breastfeeding, there are higher concentrations of PTHrP and estrogen, which is beneficial to the formation of bone mass [4]. In addition, osteocalcin and N-telopeptides are markers for bone formation, which levels increased with prolonged lactation within a certain range [37]. An approximated 6 to 12 months after weaning, the bone density is fully restored [35, 38], which may explain why the incidence of osteoporotic hip fractures has not increased in women after breastfeeding. The exact mechanism of this recovery process is unclear and needs further exploration [4].

### Limitations

Although this is the first study to use dose-response analysis to investigate the relationship between osteoporotic hip fracture and breastfeeding, the weakness of this meta-analysis should be considered. (1) There was a great difference in the sample size of included studies, especially the study of Crandall et al. [15], which may affect the accuracy of our results. (2) There may be some potential publication bias due to the limited number of studies included. (3) This meta-analysis has not been registered online in advance. (4) We did not retrieve grey literature, which may affect the accuracy of the conclusions to some extent.

## Conclusion

Our meta-analysis demonstrated that there is a non-linear relationship between breastfeeding with osteoporotic hip fracture based on the previous studies. The risk of osteoporotic hip fracture decreased with the increase of breastfeeding time. However, there is no significant relationship between them when the duration of breastfeeding time was more than 25 months.

## Data Availability

All data are fully available without restriction.

## Abbreviations

BMD: Bone mineral density
CI: Confidence interval
PTH: Parathyroid hormone
PTHrP: Parathyroid hormone-related protein
RR: Relative risk
